# Immunogenomic characteristics and prognostic implications of terminally exhausted CD8^+^ T cells in colorectal cancers

**DOI:** 10.3389/fimmu.2025.1601188

**Published:** 2025-05-30

**Authors:** Ji-Ae Lee, Hye Eun Park, Dae-Won Lee, Sae-Won Han, Tae-You Kim, Seung-Yong Jeong, Kyu Joo Park, Jeong Mo Bae, Gyeong Hoon Kang

**Affiliations:** ^1^ Department of Pathology, Seoul National University Bundang Hospital, Seoul National University College of Medicine, Seongnam-si, Gyeonggi-Do, Republic of Korea; ^2^ Laboratory of Epigenetics, Cancer Research Institute, Seoul National University College of Medicine, Seoul, Republic of Korea; ^3^ Department of Pathology, Seoul National University Boramae Hospital, Seoul National University College of Medicine, Seoul, Republic of Korea; ^4^ Department of Internal Medicine, Seoul National University Hospital, Seoul National University College of Medicine, Seoul, Republic of Korea; ^5^ Department of Surgery, Seoul National University Hospital, Seoul National University College of Medicine, Seoul, Republic of Korea; ^6^ Department of Pathology, Seoul National University Hospital, Seoul National University College of Medicine, Seoul, Republic of Korea

**Keywords:** colorectal cancer, exhausted T cell, microsatellite instability, tumor mutational burden, prognosis

## Abstract

**Introduction:**

T-cell exhaustion is a major mechanism of immune evasion. Recently, the therapeutic and prognostic implications of progenitor exhausted T cells (Tpex) and terminally exhausted T cells (Ttex) have been explored in various cancer types. This study explored the immunogenomic characteristics and prognostic implications of Tpex and Ttex in colorectal cancers (CRCs).

**Methods:**

We performed multiplex immunofluorescence (mIF) using antibodies against CK, CD3, CD8, TCF1, and FOXP3 to assess diverse subsets of tumor-infiltrating lymphocytes (TILs) in 517 patients with stage III or high-risk stage II CRCs. We compared the infiltration level of these TIL subsets with the genetic profiles of CRCs, including microsatellite instability (MSI), tumor mutational burden (TMB), and mutations in 40 tumor-associated genes across five biological pathways.

**Results:**

CD8^+^ T cell density, the CD8/CD3 ratio, and the Ttex/CD8^+^ T cell ratio were elevated in microsatellite instability-high and tumor mutational burden-high tumors. Survival analysis showed that, higher CD8^+^ T cell density, higher regulatory T cell/CD3^+^ T cell ratio, and higher Ttex/CD8^+^ T cell ratio exhibited better 5-year relapse-free survival (RFS) rates. When tumors were categorized into CD8-high, CD8-low/Ttex-low, and CD8-low/Ttex-high groups, the CD8-high and CD8-low/Ttex-high groups showed better 5-year RFS than the CD8-low/Ttex-low group.

**Discussion:**

Ttex infiltration is associated with MSI and TMB status and may serve as a prognostic marker of CRCs.

## Introduction

Colorectal cancer (CRC) is the third most common malignancy and second leading cause of cancer-related death worldwide (1). The primary treatment for CRC patients with locoregional metastasis includes curative surgery, followed by oxaliplatin-based chemotherapy. However, 20-30% of patients with stage III CRC experience tumor recurrence within 5 years (2, 3). Therefore, innovative treatment strategies are necessary to improve survival outcomes and quality of life in patients with CRC. Recent advances in immuno-oncological drug development and a better understanding of the tumor microenvironment have opened new avenues for treatment (4, 5).

T cell exhaustion is a key factor influencing the efficacy of cancer immunotherapy (6). Exhausted T cells, which arise in response to chronic antigen exposure in the tumor microenvironment, exhibit progressive loss of effector function, sustained expression of multiple inhibitory receptors such as PD1, cytotoxic T lymphocyte antigen 4 (CTLA-4), T-cell immunoglobulin domain and mucin domain protein 3 (TIM-3). and lymphocyte activation gene 3 protein (LAG-3), and impaired proliferative potential (7, 8). Immune checkpoint inhibitors, including anti-PD1 and anti-CTLA-4 therapies, have demonstrated that reversing T cell exhaustion can lead to durable clinical responses in a subset of patients (4, 9, 10).

Single-cell technologies have revealed substantial heterogeneity among exhausted T cell compartments, challenging the traditional view of T cell exhaustion as a uniform state (11, 12). Progenitor exhausted T cells (Tpex), defined by the co-expression of TCF1 and PD1 (TCF1^+^PD1^+^), are a less differentiated subset that retains self-renewal and proliferative capacity. These cells serve as reservoirs and, differentiate into more exhausted populations upon persistent antigenic stimulation. Tpex cells are characterized by high expression of co-stimulatory molecules such as CD28 and ICOS, along with intermediate levels of inhibitory receptors indicative of a partially restrained functional phenotype (13). In contrast, terminally exhausted T cells (Ttex), marked by the absence of TCF1 and expression of PD1 (TCF1^-^PD1^+^), represent a late and, highly differentiated stage of exhaustion. Ttex cells exhibit elevated expression of multiple inhibitory receptors and profound impairment in both proliferative and cytotoxic capacities (13).

Although the predictive value of PD1^+^ CD8^+^ T cells for immune checkpoint blockade (ICB) treatment is well-established (14–19), the predictive value of Tpex or Ttex for ICB treatment remains controversial (20–22). Interestingly, Escobar et al. reported that the role of TCF1 expression in ICB responsiveness may depend on tumor antigenicity (23). Therefore, the clinical significance of Tpex and Ttex in CRCs requires further investigation.

From a molecular perspective, microsatellite instability (MSI) and tumor mutational burden (TMB) have emerged as predictive biomarkers for ICB therapy. MSI results from defects in the mismatch repair system, leading to frameshift mutations, and elevated the neoantigen loads (24). TMB is a measure of the total number of mutations per coding area of the tumor genome (25, 26). Although CRCs with MSI or high TMB are known to harbor increased tumor-infiltrating lymphocytes (TILs), the degree of infiltration and the proportion of diverse TIL subsets according to MSI and TMB status have not been thoroughly explored.

Quantitative measurement of TILs in routine hematoxylin and eosin (H&E) stained slides may offer a cost-effective approach, particularly with recent advances in digital pathology and artificial intelligence. However, the heterogeneity of diverse TIL subsets could not be evaluated using H&E staining. The utility of the Immunoscore^®^, which is based on the quantification of the total CD3^+^ T lymphocyte and CD8^+^ cytotoxic T lymphocyte (CTL) populations, has been validated in several studies (27–29). However, the conventional Immunoscore^®^ method requires two glass slide sections and cannot measure the functional state of CTLs. Multiplex imaging enables the simultaneous detection of multiple surface antigens within a single tissue section. Therefore, multiplex imaging has become a powerful method for quantitative analysis of the tumor immune microenvironment.

In this study, we employed multiplex immunofluorescence (mIF) to analyze T-cell exhaustion in patients with CRC who underwent curative surgery followed by adjuvant oxaliplatin-based chemotherapy. We evaluated the genomic and immunological correlations in CRCs by combining molecular characteristics, including MSI and TMB status. Moreover, we aimed to identify whether the presence of Ttex in tumor tissues is associated with the clinical outcomes of patients with CRC.

## Materials and methods

### Study populations

Initially, 655 patients with stage III or high-risk stage II CRCs received adjuvant fluoropyrimidine plus oxaliplatin after curative (R0) resection at the Seoul National University Hospital between April 2005 and December 2012 were selected for this study (30). After reviewing tissue availability for tissue microarray (TMA) construction, tumor samples from 517 patients were subjected to mIF analysis.

MSI was assessed at the following loci as previously described (31): BAT25, BAT26, D2S123, D5S346, and D17S250. The CpG island methylator phenotype (CIMP) subtype was evaluated using the MethyLight assay. DNA methylation was quantified using the following eight CIMP panel markers: *CACNA1G, CDKN2A, CRABP1, IGF2, MLH1, NEUROG1, RUNX3*, and *SOCS1*. The primer sequences and polymerase chain reaction conditions have been described previously.

### TMA construction

After microscopic examination of H&E-stained slides from formalin-fixed paraffin-embedded tissues, four tissue cores (1 mm in diameter) from the tumor center (TC) and invasive margin (IM) (two cores each for TC and IM) for each case were sampled and transferred to TMA blocks. Tertiary lymphoid structures identified by microscopic examination were avoided because they might be independent biomarkers and showed Tpex abundance.

### mIF staining

mIF staining, scanning, and analysis were performed by prismCDX Co. Ltd. (Gyeonggi-do, Republic of Korea). Specimen sections (4 μm thick) were cut from the TMA blocks. Slides were heated for at least 1 h in a dry oven at 60°C, followed by mIF staining with a Leica Bond Rx™ Automated Stainer (Leica Biosystems, Wetzlar, Germany). The antibodies and fluorophores used are listed in **Supplementary Table S1**. Briefly, slides were dewaxed with Leica Bond Dewax solution (#AR9222, Leica Biosystems), followed by antigen retrieval using Bond Epitope Retrieval 2 (#AR9640, Leica Biosystems) for 30 min. Staining was performed in sequential rounds of blocking buffer (#C0103, TheraNovis, Seoul, Republic of Korea), followed by incubation with the primary antibody for 30 min, and incubation with mouse or rabbit HRP-conjugated secondary antibody (#C0105, TheraNovis) for 10 min. The antigen was visualized using Astra-dye (TheraNovis) for 10 min, after which the slide was treated with Bond Epitope Retrieval 1 (#AR9961, Leica Biosystems) for 20 min to remove the bound antibodies before the next step. The process from blocking to antigen retrieval was repeated for each antibody staining. Nuclei were counterstained with DAPI (#C0106, TheraNovis) after the last round of antigen retrieval. The slides were coverslipped using the ProLong Gold antifade reagent (P36930, Invitrogen, Carlsbad, CA, USA).

### Multispectral imaging and analysis

Multiplex stained slides were scanned using PhenoImager™ (Akoya Biosciences, Marlborough, MA, USA) at 20× magnification. Representative images for training were selected using Phenochart™ Whole Slide Viewer (version 1.0.12, Akoya Biosciences), and an algorithm was created in the inForm Tissue Analysis software (version 2.6, Akoya Biosciences). Multispectral images were unmixed using the spectral library of the inForm software, and the tumor tissue was segmented according to the presence or absence of CK expression. Individual cells were segmented based on DAPI staining, and phenotyping was performed according to the expression compartment and intensity of each marker. After designating the region of interest to be analyzed on the tissue slides, the same algorithm was batch-applied. The exported data were consolidated and analyzed in R studio (version 4.2.1; R Foundation for Statistical Computing, Vienna, Austria) using the phenoptr and phenoptrReports packages (Akoya Biosciences). Owing to tissue detachment and folding, 517 patients with TC cores and 505 patients with IM cores were analyzed.

### Quantification of TILs using whole-slide imaging

The mean CD3^+^ and CD8^+^ TIL densities were obtained from a previous study (32). Briefly, immunohistochemistry was performed using antibodies against CD3 (1:300, Dako, Santa Clara, CA, USA) and CD8 (SP57, ready-to-use, Ventana Medical Systems, Oro Valley, AZ, USA) in representative tumor sections. All staining procedures were conducted using a Ventana BenchMark XT system according to the manufacturer’s protocol. The slides were scanned using an Aperio AT2 slide scanner (Leica Biosystems) at 20× magnification. TILs were quantified using a custom algorithm in the QuPath software.

### Targeted sequencing of 40 genes and TMB calculation

Among the 655 patients with stage III or high-risk stage II CRCs, 516 underwent targeted sequencing. Briefly, 40 genes associated with five critical pathways were sequenced using HiSeq 2500, as previously described (33). We selected 14 genes from the WNT pathway (*ARID1A, AMER1, APC, AXIN2, CTNNB1, DKK1, DKK2, DKK3, DKK4, FBXW7, FZD10, LRP5, SOX9*, and *TCF7L2*), 2 genes from the P53 pathway (*ATM* and *TP53*), 8 genes from the RTK-RAS pathway (*BRAF, EGFR, ERBB2, ERBB3, ERBB4, HRAS, KRAS*, and *NRAS*), 7 genes from the TGFβ pathway (*ACVR1B, ACVR2A, SMAD2, SMAD3, SMAD4, TGFBR1*, and *TGFBR2*), and 9 genes from the PI3K pathway (*IGF1R, IGF2, IRS2, MTOR, PDGFRA, PIK3CA, PIK3R1, PTEN*, and *SRC*). TMB was calculated using the total number of non-synonymous somatic mutations. Tumors were classified as TMB-high if the tumor tissue contained eight or more mutations. A total of, 463 cases with both targeted sequencing and mIF data were subjected to statistical analysis for immunogenomic characterization.

### Statistical analysis

All statistical analyses were performed in R. Clinicopathologic characteristics between MSS-CRCs and MSI-CRCs were compared using χ^2^-test for categorical variables and Wilcoxon’s rank-sum test for ordinal variables. For survival analysis, Kaplan-Meier curves were plotted, and the Cox proportional hazard model was used for univariate and multivariate analyses. To determine the optimal cut-off value for survival analysis, maximally selected rank statistics were employed using the *maxstat* R package (34). Based on the cut-off value, all samples with a nonzero denominator were classified into a high or a low group. All statistical tests were two-sided, and statistical significance was defined as *P* < 0.05.

## Results

### Correlation between TIL subsets in the TC and IM

**Figure 1:**
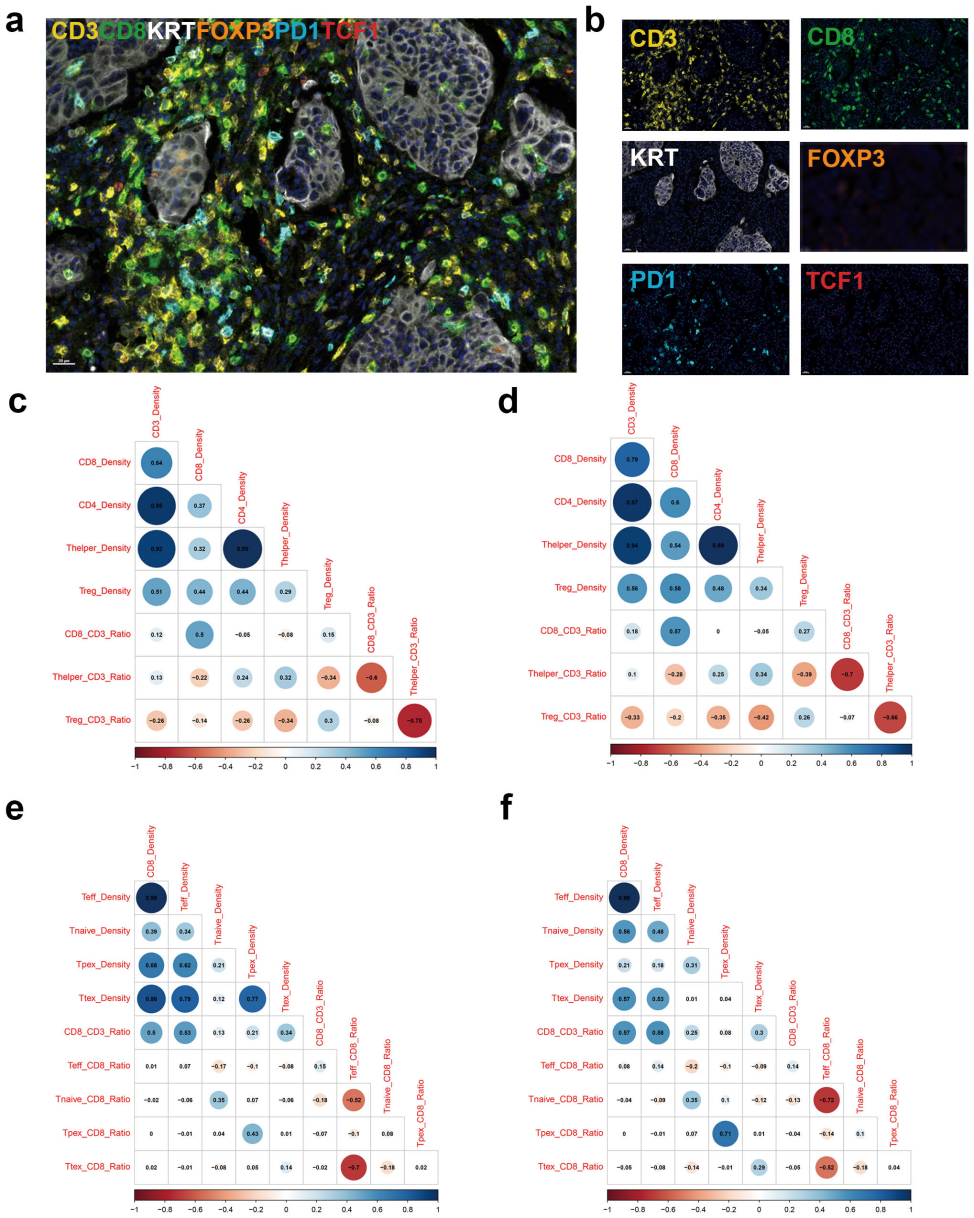
Multiplex immunofluorescence stains focusing on tumor-infiltrating lymphocytes. **(a)** Representative image of merged multiplex immunofluorescence, **(b)** immunofluorescence image for individual markers, **(c)** Corrplot for showing density and ratio of major T cell types in the tumor center (TC), **(d)** Corrplot for density and ratio of major T cell types in the invasive margin (IM), **(e)** Corrplot for density and ratio of CD8^+^ T cell subsets in the TC, and **(f)** Corrplot for density and ratio of CD8^+^ T cell subsets in the IM.

mIF staining focusing on T cells was performed in 517 patients with stage III or high-risk stage II CRC (**Table 1**, **Supplementary Table S2**). Representative multispectral images of mIF of CD3, CD8, CK, FOXP3, PD1, and TCF1 are shown in **Figures 1a, b**. We first compared CD3^+^ and CD8^+^ TIL density between WSI and mIF using TMA to examine whether the four tissue cores (two cores each of TC and IM) were representative. Although different antibody clones were used for the WSI and mIF assays, the density of CD3^+^ TILs in the TC (Pearsons’s rho: 0.46, *P* < 2.2×10^-16^), CD8^+^ TILs in the TC (Pearson’s rho: 0.6, *P* < 2.2×10^-16^), and IM (Pearson’s rho: 0.51, *P* < 2.2×10^-16^) showed moderate correlations between WSI and TMA, whereas the CD3^+^ TIL density in the IM showed a weak correlation (Pearson’s rho: 0.34, *P* = 1.5×10^-13^) between WSI and TMA (**Supplementary Figure S1**). We then analyzed the correlation between the major T cell subsets. The density of CD8^+^ TILs was weakly correlated with that of CD4^+^ T cells, helper T cells (Thelper), and regulatory T cells (Treg) in the TC (**Figure 1c**); however, these correlations were stronger in the IM (**Figure 1d**). Interestingly, the CD8/CD3 ratio was negatively correlated with helper T cell/CD3 and regulatory T cell/CD3 ratios in both the TC and IM (**Figures 1c, d**). We then analyzed correlation between different CTL subtypes. In TC, Ttex density was strongly correlated with effector T cell density and Tpex density (**Figure 1e**). In the IM, Ttex density was moderately correlated with effector T cell density but was not correlated with Tpex density (**Figure 1f**). Intriguingly, the Ttex/CD8 ratio showed a strong negative correlation with the effector T cell/CD8 ratio in TC and a moderate negative correlation in IM (**Figures 1e, f**)

**Table 1:** Demographic data of enrolled patients.

Variable	MSS-CRCs (n = 479, 92.6%)	MSI-CRCs (n = 38, 7.4%)	*P*
Age (years)	60 (29–78)	55 (33–78)	0.030
Sex			0.094
Male	287 (91.1%)	28 (8.9%)	
Female	192 (95.0%)	10 (5.0%)	
Location			<0.001
Proximal colon	147 (84.5%)	27 (15.5%)	
Distal colon	271 (96.1%)	11 (3.9%)	
Rectum	61 (12.7%)	0 (0.0%)	
Gross pattern			0.605
Fungating	282 (92.2%)	24 (7.8%)	
Ulcerative	197 (93.4%)	14 (6.6%)	
Differentiation			<0.001*
Well differentiated	23 (95.8%)	1 (4.2%)	
Moderately differentiated	431 (94.3%)	26 (5.7%)	
Poorly differentiated	25 (69.4%)	11 (30.6%)	
T category			0.841*
T2	22 (95.6%)	1 (4.4%)	
T3	380 (92.5%)	31 (7.5%)	
T4	77 (92.8%)	6 (15.8%)	
N category			0.041*
N0	74 (88.1%)	10 (11.9%)	
N1	274 (92.6%)	22 (7.4%)	
N2	131 (95.6%)	6 (4.4%)	
Stage			0.081
II, high-risk	74 (88.1%)	10 (11.9%)	
III	405 (93.5%)	28 (6.5%)	
TMB (N = 377)			<0.001
TMB-low (< 8)	326 (97.9%)	7 (2.1%)	
TMB-high (≥ 8)	22 (50.0%)	22 (50.0%)	

*Wilcoxon’s rank-sum test.

CRC, colorectal cancer; MSI, microsatellite instability; MSS, microsatellite stable; TMB, tumor mutational burden.

### Densities and fractions of major T cell subsets according to the mutations in critical pathway genes

Previously, we sequenced 40 genes involved in five key pathways in CRC (33). At the individual gene level, *ATP* and *TP53* mutations were common in MSS CRCs, whereas *PIK3CA*, *TCF7L2*, *SOX9*, and *ARID1A* mutations were frequently observed in MSI CRCs (**Supplementary Table S3**). *APC* mutations were linked to reduced CD8^+^ T cell density, Treg density, CD8/CD3 ratio, and Treg/CD3 ratio. Conversely, *ARID1A* mutations were associated with increased CD8^+^ T cell density and CD8/CD3 ratio (**Supplementary Tables S4**-**S10**).

**Figure 2:**
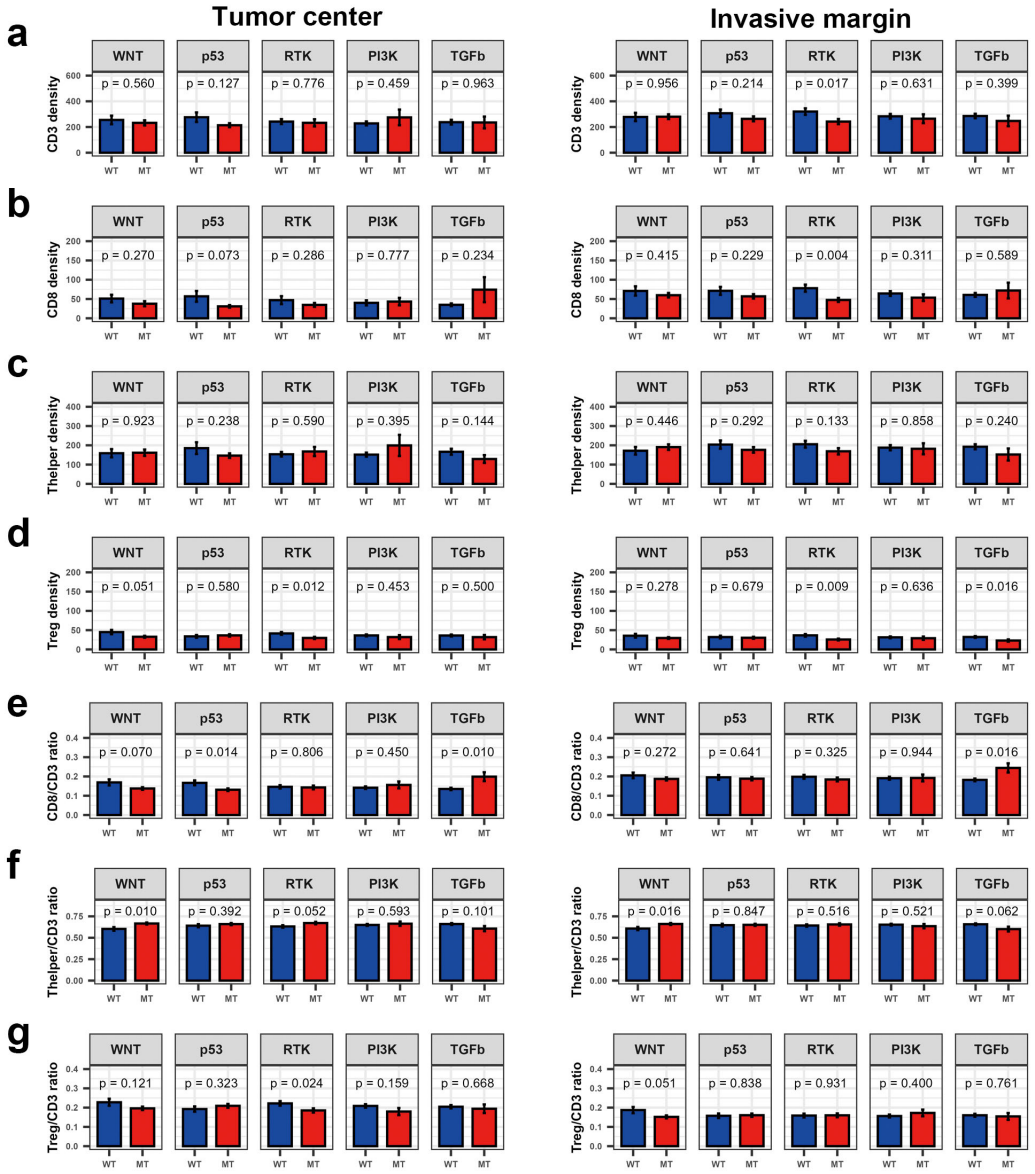
Bar plot showing density and fraction of major T cell types according to the mutations in five molecular pathways in colorectal cancer. **(a)** CD3^+^ T cell density, **(b)** CD8^+^ T cell density, **(c)** Thelper density, **(d)** Treg density, **(e)** CD8/CD3 ratio, **(f)** Thelper/CD3 ratio, and **(g)** Treg/CD3 ratio. ns, *P* > 0.05; *P ≤ 0.05 (Wilcoxon’s rank-sum test).

At the pathway level, p53 pathway mutations were prevalent in MSS CRCs, while mutations in the PI3K and TGFβ pathways were more frequent in MSI CRCs (**Supplementary Table S11**). Mutations in the WNT pathway were associated with an increased Thelper/CD3 ratio in both the TC and IM (**Figure 2**). Mutations in the RTK pathway were linked to reduced Treg density in both TC and IM; however, an association between RTK pathway mutations and a low Treg/CD3 ratio was observed only in TC. Additionally, mutations in the TGFβ pathway correlated with a higher CD8/CD3 ratio in both TC and IM.

Mutations in individual genes rarely affected the proportions of each CTL subtype (data not shown). TGFβ pathway mutations were associated with a lower Tnaïve/CD8 ratio and Tpex/CD8 ratio in the TC, whereas the Ttex/CD8 ratio did not differ significantly based on TGFβ pathway mutation status (**Supplementary Figure S2**).

### Densities of major T cell subsets and proportions of CTL subtypes according to the MSI and TMB statuses

Tumors with MSI and high TMB have a high number of TILs, owing to increased neoantigenicity. To determine which T cell subsets were affected by MSI and TMB status, we first compared the major T cell subsets according to the MSI status. In both TC and IM, the densities of CD3^+^, CD8^+^, and helper T cells were significantly higher in MSI tumors than in microsatellite stable (MSS) tumors (**Supplementary Figures S3a, c**). Correspondingly, TMB-high tumors had higher CD8^+^ T cell densities than TMB-low tumors (**Supplementary Figures S3b, d**). However, TMB-high and TMB-low tumors showed equivalent densities of both CD3^+^ T cells and helper T cells (**Supplementary Figures S3b, d**). Regulatory T cell density was not significantly associated with MSI or TMB statuses.

**Figure 3:**
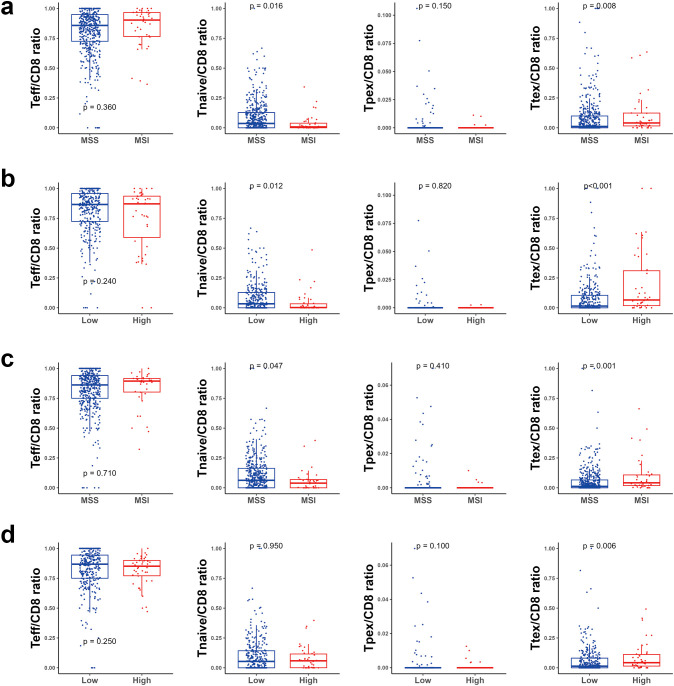
Bar plot for cytotoxic T cell subsets. **(a)** according to the microsatellite instability (MSI) status in the tumor center (TC), **(b)** according to the tumor mutational burden (TMB) status in the TC, **(c)** according to the MSI status in the invasive margin (IM), and **(d)** according to the TMB status in the IM. ns, *P* > 0.05; *P ≤ 0.05, **P ≤ 0.01; ***P ≤ 0.001 (Wilcoxon’s rank-sum test).

Next, we explored the CTL subtypes in more detail in relation to MSI and TMB status. In TC, neither the effector T cell/CD8 ratio nor Tpex/CD8 ratio were significantly different between MSI and MSS tumors. However, a lower naïve T cell/CD8 ratio and higher Ttex/CD8 ratio were observed in MSI tumors. However, a lower naïve T cell/CD8 ratio and higher Ttex/CD8 ratio were observed in MSI tumors (**Figure 3a**). Likewise, TMB-high tumors showed a decreased naïve T cell/CD8 ratio and an increased Trex/CD8 ratio compared with TMB-low tumors (**Figure 3b**). In IM, the Ttex/CD8 ratio was significantly higher in MSI and TMB-high tumors than in MSS and TMB-low tumors (**Figures 3c, d**).

### Association between diverse T cell parameters and patient survival

**Figure 4:**
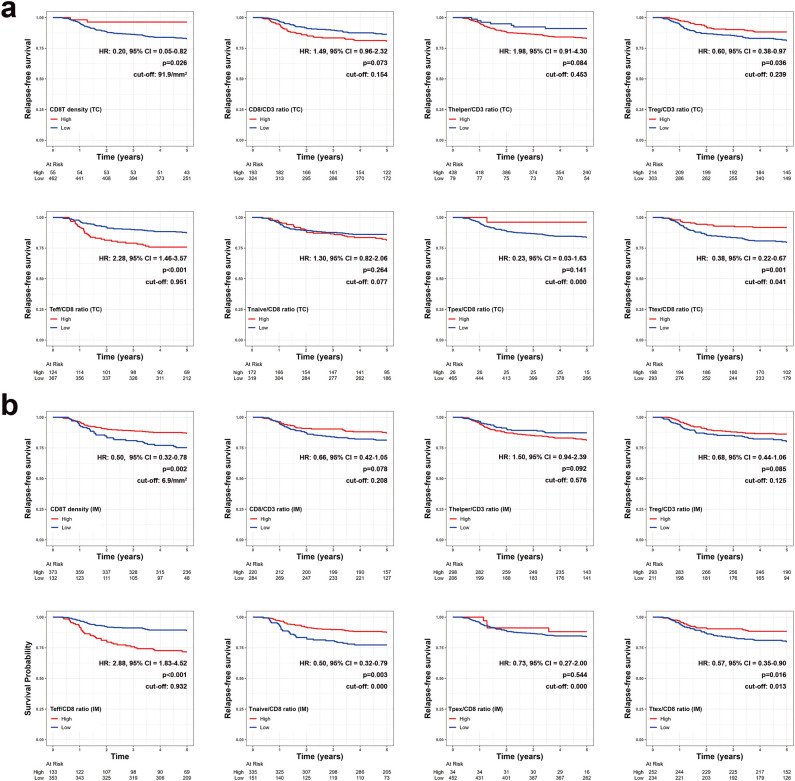
Kaplan-Meier survival curves for 5-year relapse-free survival based on various T cell parameters in the **(a)** tumor center (TC) and **(b)** invasive margin (IM). Patients with zero denominator were excluded in survival curves.

Survival analyses were performed to identify the prognostic impact of each T cell parameter. In TC, univariate analysis using Kaplan-Meier survival curves showed that the 5-year relapse-free survival (RFS) rate was significantly higher in groups with high CD8^+^ T cell density (*P* = 0.026) and a high Ttex/CD8 ratio (*P* = 0.001) (**Figure 4a**). The group with a high Treg/CD3 ratio also exhibited a higher 5-year RFS rate than that with a low Treg cell/CD3 ratio (*P* = 0.036). In contrast, the group with a high Teff/CD8 ratio showed a lower 5-year RFS rate than that with lower Teff/CD8 ratio (*P* < 0.001).

Similarly, in IM, 5-year RFS rates were significantly higher in patients with high CD8^+^ T cell density (*P* = 0.002), a high Ttex/CD8 ratio (*P* = 0.016), and significantly lower in those with a high Teff/CD8 ratio groups (*P* < 0.001) (**Figure 4b**). A high Treg/CD3 ratio in the IM was not significantly associated with a better 5-year RFS. However, the Thelper/CD3, Tnaïve cell/CD8 ratio, and Ttex/CD8 ratio were not significantly associated with the 5-year RFS in either TC and IM.

### Prognostic role of Ttex infiltration in patients with CRC

**Figure 5:**
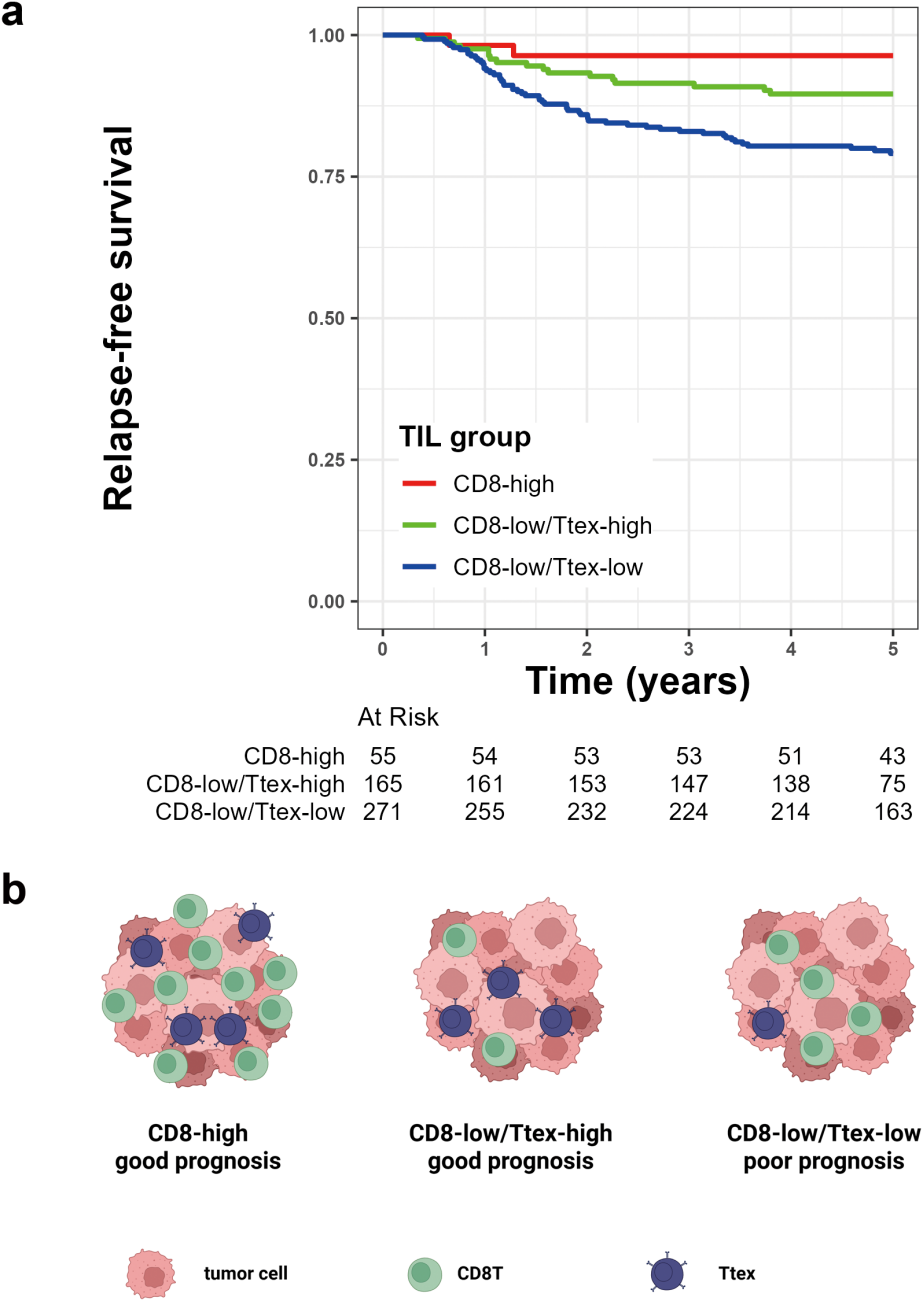
Prognostic value of tumor-infiltrating lymphocytes (TIL) group based on CD8^+^ T cell infiltration and terminally exhausted CD8^+^ T cell (Ttex) fraction. **(a)** Kaplan-Meier survival curve for 5-year relapse-free survival, and **(b)** graphical summary.

Finally, we categorized the patients with CRC into three groups based on the amount of CD8^+^ T cell infiltration and exhaustion status. We first categorized the patients into CD8-high and CD8-low groups according to their CD8 density in the TC. The CD8-low group was further divided into CD8-low/Ttex-high and CD8-low/Ttex-low groups based on the TCF1^-^PD1^+^CD8^+^ T cell/total CD8^+^ T cell ratio. The CD8^+^ T cell density was not significantly different between the CD8-low/Ttex-low and CD8-low/Ttex-high groups (**Supplementary Figure S4**). As expected, the CD8-high group showed the best prognosis, followed by the CD8-low/Ttex-high and CD8-low/Ttex-low groups (**Figure 5a**).

In our previous study, we demonstrated that TMB was more strongly associated with CRC prognosis than the MSI status (33). Given that the degree of Ttex infiltration was significantly correlated with both TMB and MSI, we evaluated the potential interactions between TIL groups and these molecular features. The p-value for the interaction between TIL group and TMB was 0.261, whereas the p-value for the TIL group and MSI status was 0.326, indicating no significant interaction effects. After adjusting for clinicopathological variables, multivariate Cox regression analyses revealed at the TIL group was an independent predictor of the 5-year RFS (**Table 2**). The graphical summary of this study is presented in **Figure 5b**.

**Table 2:** Multivariate survival analysis.

Variable	Status	Univariate analysis		Multivariate analysis	
		HR (95% CI)	*P*	HR (95% CI)	*P*
T category	T2,3	1		1	
	T4	2.19 (1.34–3.58)	0.002	1.97 (1.20–3.23)	0.007
N category	N0,1	1		1	
	N2	2.44 (1.57–3.79)	<0.001	1.93 (1.22–3.03)	0.005
Lymphatic invasion	Absent	1		1	
	Present	2.72 (1.72–4.31)	<0.001	2.42 (1.51–3.88)	<0.001
TIL group	CD8-low/T_tex_-low	1		1	
	CD8-low/T_tex_-high	0.48 (0.28–0.83)	0.008	0.55 (0.32–0.94)	0.029
	CD8-high	0.16 (0.04-0.66)	0.011	0.16 (0.04–0.66)	0.011
Venous invasion	Absent	1			
	Present	1.92 (1.09–3.36)	0.023		ns
Perineural invasion	Absent	1			
	Present	2.08 (1.34–3.24)	0.001		ns
TMB	Low	1			
	High	0.17 (0.04-0.69)	0.014		ns
MSI status	MSS	1			
	MSI	0.84 (0.34-2.08)	0.707		ns

CI, confidence interval; HR, hazard ratio; TIL, tumor-infiltrating lymphocyte, T_tex_, terminally exhausted CD8^+^ T cells; TMB, tumor mutational burden; MSI, microsatellite instability; MSS, microsatellite stable; ns, not significant.

## Discussion

Tumor-infiltrating T cells are associated with patients’ survival, and as reflected by the Immunoscore^®^, tumor-infiltrating T cells including CTLs are important for predicting the outcome in patients with CRC. Various subsets of T cells that infiltrate the tumor microenvironment and discriminate between tumor-reactive and bystander T cells may help select patients who would respond to immunotherapy and predict patient’ survival. Tumor-reactive T cells in the tumor microenvironment show an exhausted phenotype, expressing inhibitory cell surface markers such as PD1, LAG-3, and TIM-3. The infiltration of exhausted T cells into the tumor microenvironment can be interpreted as a tumor-specific T cell response, and owing to the expression of PD1, these cells are potential direct targets for anti-PD1 therapies. Therefore, the predictive and prognostic roles of PD1^+^CD8^+^ T cells in ICB treatment have been widely studied in patients with CRC (16–19).

However, Heterogeneity exists within exhausted TILs, including Tpex to Ttex. These diverse subsets of exhausted TILs have different effects on the responsiveness to immunotherapy. Tpex, marked by TCF1 expression, is known for its self-renewal capacity and the ability to differentiate into T cell populations with effector functions. Previous studies have reported that Tpex undergo substantial expansion in response to PD1 blockade therapy and that this T cell subset can be a target for immunotherapy (11). Tumor-resident Tpex is preferentially located in tertiary lymphoid structures, providing a niche to support anti-tumor immunity (35, 36). In CRC, Crohn-like lymphoid reaction (CLR) represents tertiary lymphoid structures and serves as an independent prognostic factor. In the present study, we excluded the CLR areas when selecting representative tumor cores to minimize their prognostic influence. In our analysis, Tpex was sparsely distributed in the tumor areas of both TC and IM. Despite the high expression of terminal exhaustion markers, Ttex plays a distinct role in tumor immunity. Although Ttex has been thought to be dysfunctional, recent studies have demonstrated that Ttex express cytotoxic molecules such as granzymes, perforin, and interferon γ (37). This implies that some Ttex retain their effector function. Recently, Chen et al. analyzed the changes in the tumor microenvironment before and after ICB therapy using single-cell RNA sequencing in 22 patients with CRC. In patients who achieved complete remission, high Ttex infiltration was observed before treatment, which was followed by replacement with Tpex after therapy. In contrast, patients in partial remission exhibit low Ttex infiltration at baseline, which increases after treatment (38).

Oxaliplatin-based combination chemotherapy is the standard adjuvant treatment after surgical resection in patients with locally advanced CRCs (39, 40). Oxaliplatin, a platinum-based cytotoxic agent, exerts its anti-tumor effects primarily by inducing DNA crosslinking, thereby disrupting DNA replication and transcription (41, 42). In addition to its direct cytotoxicity against tumor cells, oxaliplatin modulates the tumor microenvironment by inducing immunogenic cell death (43, 44). Immunogenic cell death promotes dendritic cell maturation, enhances antigen presentation, and primes tumor-specific T cells, thereby restoring anti-tumor immune responses within the tumor microenvironment. The improved clinical outcomes observed in CRC patients with a high Ttex/CD8 ratio, may be partially attributable to the immunogenic effects induced by oxaliplatin.

MSI and TMB statuses can affect the robustness of antitumor immunity by modulating immune cell infiltration. We investigated whether MSI and TMB status could affect the composition of TILs, particularly the exhausted phenotype. In our analysis, MSI and TMB-high tumors showed higher CTL densities and higher Ttex/CD8 ratio in both TC and IM compared with MSS and TMB-low tumors. These findings suggest that tumors with a high neoantigen load can not only attract CTLs into the tumor, but that tumor-reactive T cells, especially Ttex, are also highly present in the infiltrates.

However, the prognostic value of Tpex and Ttex in CRCs have not yet been widely evaluated. Huang et al. and Tran et al. evaluated the proportion of Tpex in CRCs using mIF, and reported that patients with CRC with increased Tpex levels showed better clinical outcomes (45, 46). However, Huang et al.’s study included 104 CRC patients without stage limitations, and mIF analysis was performed only in 63 patients with stage III CRC in Tran et al.’s study. In our survival analyses of 517 patients with stage III or high-risk stage II CRC, the proportion of Tpex was not associated with clinical outcomes in either the TC or IM. Instead, the high Ttex/CD8 ratio group showed better 5-year RFS than the low Ttex/CD8 ratio group. We further divided tumors with low CD8^+^ T cell density into CD8-low/Ttex-high and CD8-low/Ttex-low groups based on the TCF1^-^PD1^+^CD8^+^ T cell/total CD8^+^ T cell ratio. According to this analysis, the CD8-low/Ttex-high group had better RFS than the CD8-low/Ttex-low group. Our data suggest that Ttex has a beneficial influence on patient survival, and it is possible to analyze patients’ prognosis more precisely by integrating both CTL density and the Ttex/CD8 ratio.

In the current era of widespread ICB use, there is an increasing need for routine pathological diagnostics for the precise and quantitative assessment of immune cell infiltration and their functional status within the tumor microenvironment to predict prognosis, guide therapeutic strategies, and evaluate responses to ICB treatment. mIF has emerged as a powerful and relatively cost-effective method that enables the simultaneous detection of multiple biomarkers within a single tissue section while preserving the spatial architecture (47, 48). This technique facilitates comprehensive profiling of the tumor microenvironment, including the identification of specific immune cell subsets and evaluation of immunoregulatory protein expression. The integration of mIF with digital pathology platforms enhances the objectivity, reproducibility, and efficiency of immune profiling (49). The incorporation of these technologies into routine pathology workflows is expected to support personalized therapeutic decision-making and improve clinical outcomes in patients with cancer.

Our study has a few limitations. First, we categorized the effector T cells, naïve T cells, Tpex, and Ttex based on their TCF1 and PD1 expression statuses. Ttex expresses many inhibitory receptors, including PD1, LAG-3, TIM-3, CTLA-4, and T-cell immunoglobulin and immunoreceptor tyrosine-based inhibitory motif domain (TIGIT). Since the timing of their expression and their clinical implications exhibit subtle differences, using PD1 alone as an exhaustion marker is insufficient for distinguishing Ttex (50). Furthermore, identifying effector T cells as TCF1^-^PD1^-^CD8^+^ T cells without considering the expression of cytotoxic molecules or cytokines may not fully reflect effector function. However, using multiplex images, we successfully analyzed the CTL subsets and determined the prognostic impact of Ttex. Secondly, this study was conducted at a single institution and had a retrospective design. Further studies are required to investigate the association between the Ttex and therapeutic responsiveness in patients undergoing ICB therapy.

In conclusion, Ttex infiltration in patients with stage III or high-risk stage II CRC treated with curative surgery, followed by adjuvant oxaliplatin-based chemotherapy, was associated with favorable outcomes. This TIL population was also related to the MSI and TMB status.

## Data Availability

The datasets presented in this article are not readily available due to the retrospective nature and consent limitations. This study was conducted retrospectively, and at the time of patient enrollment, informed consent did not include provisions for public data sharing or secondary use of data. Requests to access the datasets should be directed to the corresponding author(s).
