# Supplementary material for: Immunogenomic characteristics and prognostic implications of terminally exhausted CD8+ T cells in colorectal cancers
Source: Front Immunol. 2025 May 30;16:1601188. doi: 10.3389/fimmu.2025.1601188 (PMC12163321; doi:10.3389/fimmu.2025.1601188)
Supplement: Supplementary file 1 [file DataSheet1.docx]

**
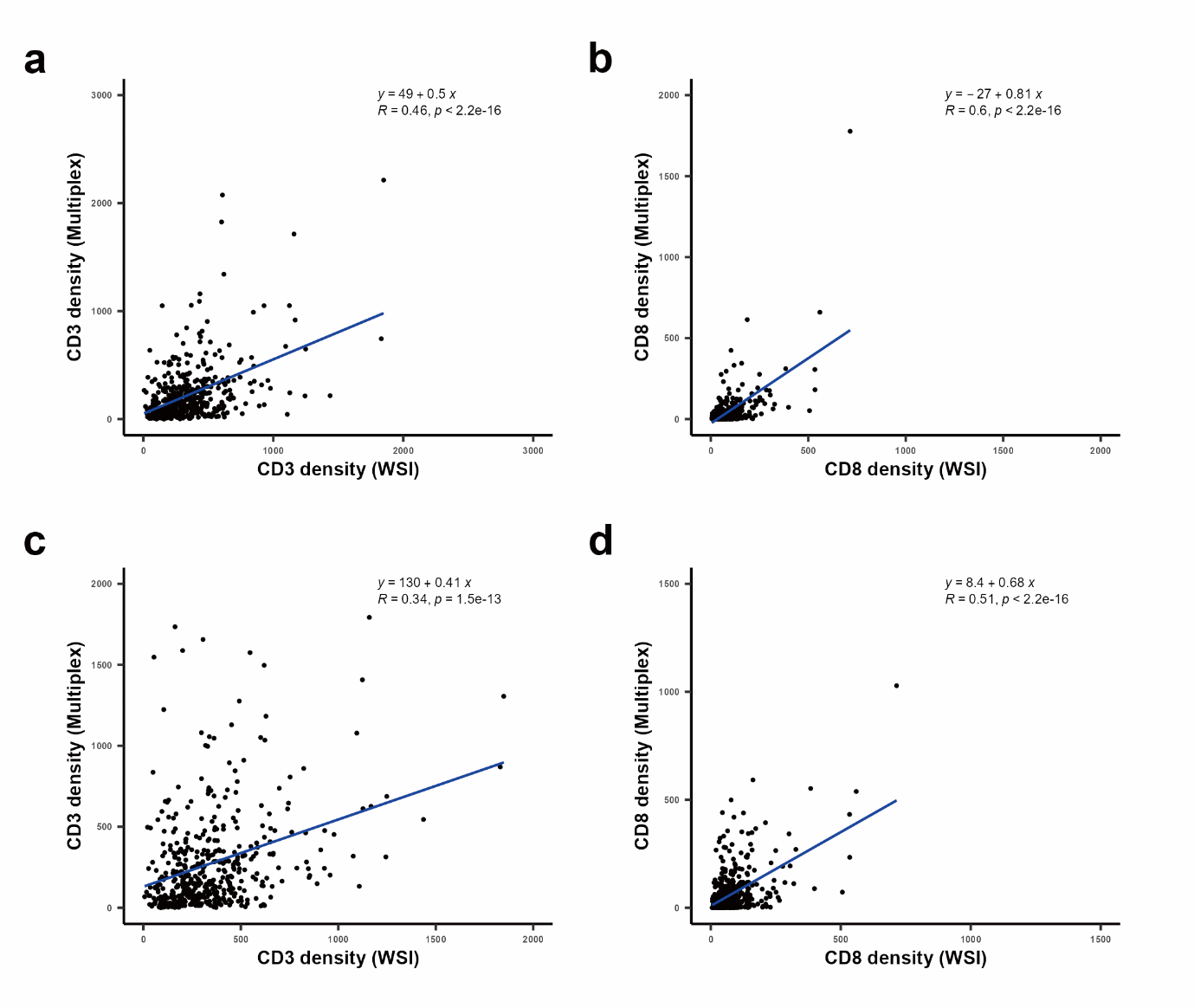
**

**Supplementary Figure 1.** Scatter plot comparing T cell infiltration between whole slide imaging (WSI) and multiplex immunofluorescence stain in the tissue microarray (TMA). (a) CD3^+^ T cell density in the tumor center (TC), (b) CD8^+^ T cell density in the TC, (c) CD3^+^ T cell density in the invasive margin (IM), and (d) CD8^+^ T cell density in the IM.


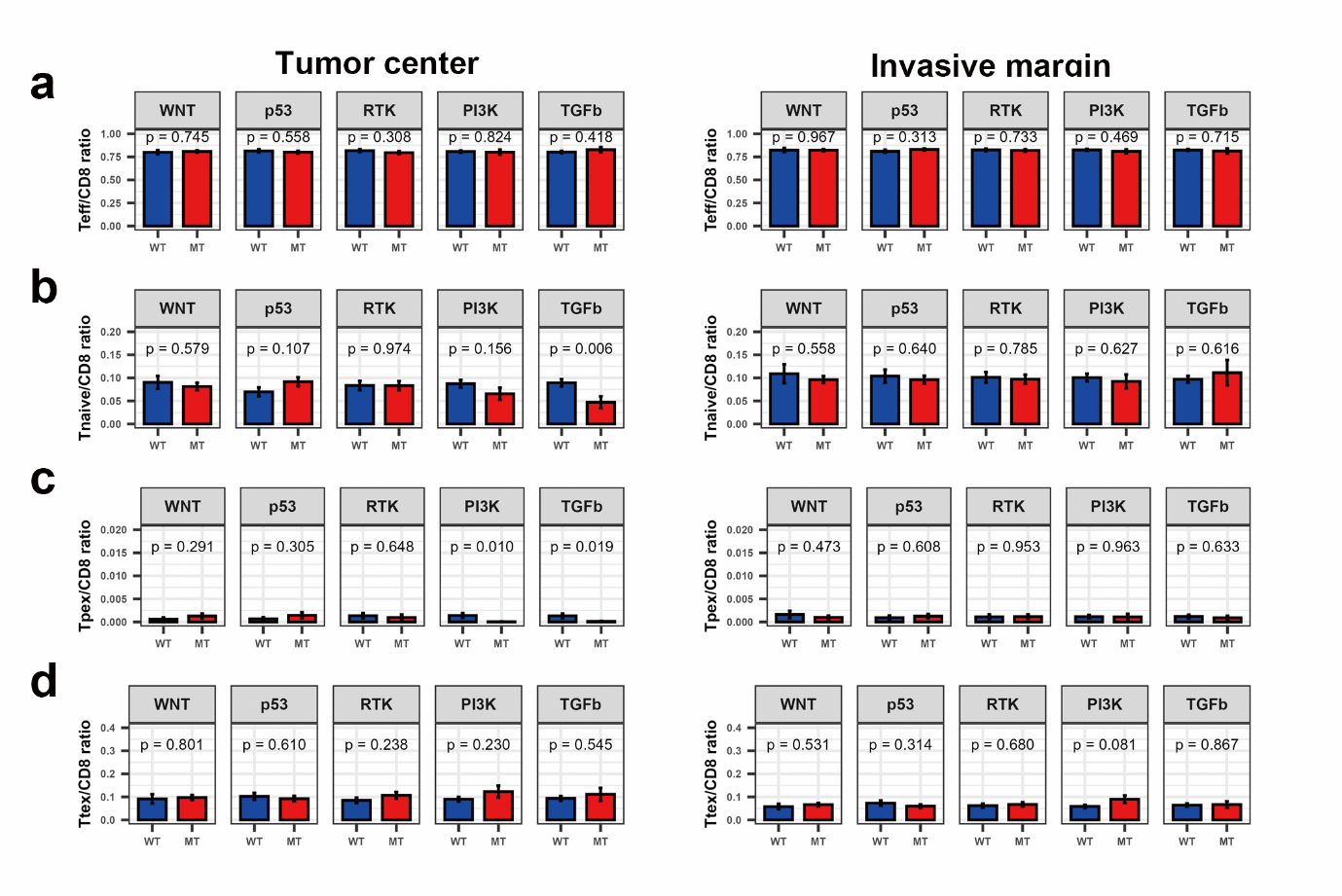


**Supplementary Figure 2.** Bar plot showing the fractions of CD8^+^ T cell subsets according to mutations in five molecular pathways in colorectal cancer. (a) Teff/CD8 ratio, (b) Tnaïve/CD8 ratio, (c) Tpex/CD8 ratio, and (d) Ttex/CD8 ratio. ns, *P* > 0.05; * *P* ≤ 0.05 (Wilcoxon’s rank-sum test).


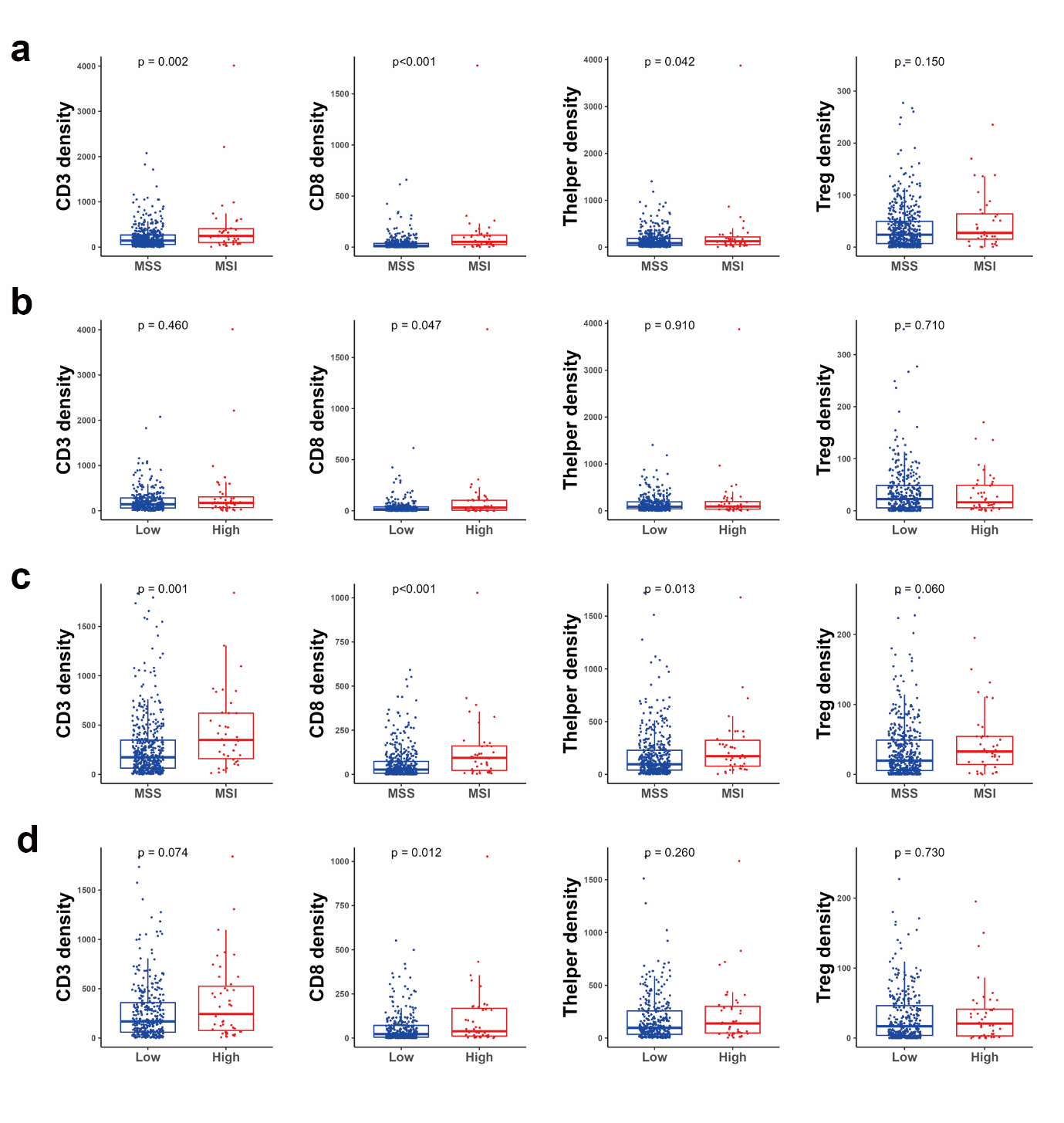


**Supplementary Figure 3**. Box plot showing the density of major T cell types according to microsatellite instability (MSI) status and tumor mutational burden (TMB). (a) According to the MSI status in the tumor center (TC), (b) according to the TMB status in the TC, (c) according to the MSI status in the invasive margin (IM), and (d) according to the TMB status in the IM. ns, *P* > 0.05; * *P* ≤ 0.05, **, *P* ≤ 0.01; ***, *P* ≤ 0.001; ****, *P* ≤ 0.0001 (Wilcoxon’s rank-sum test).


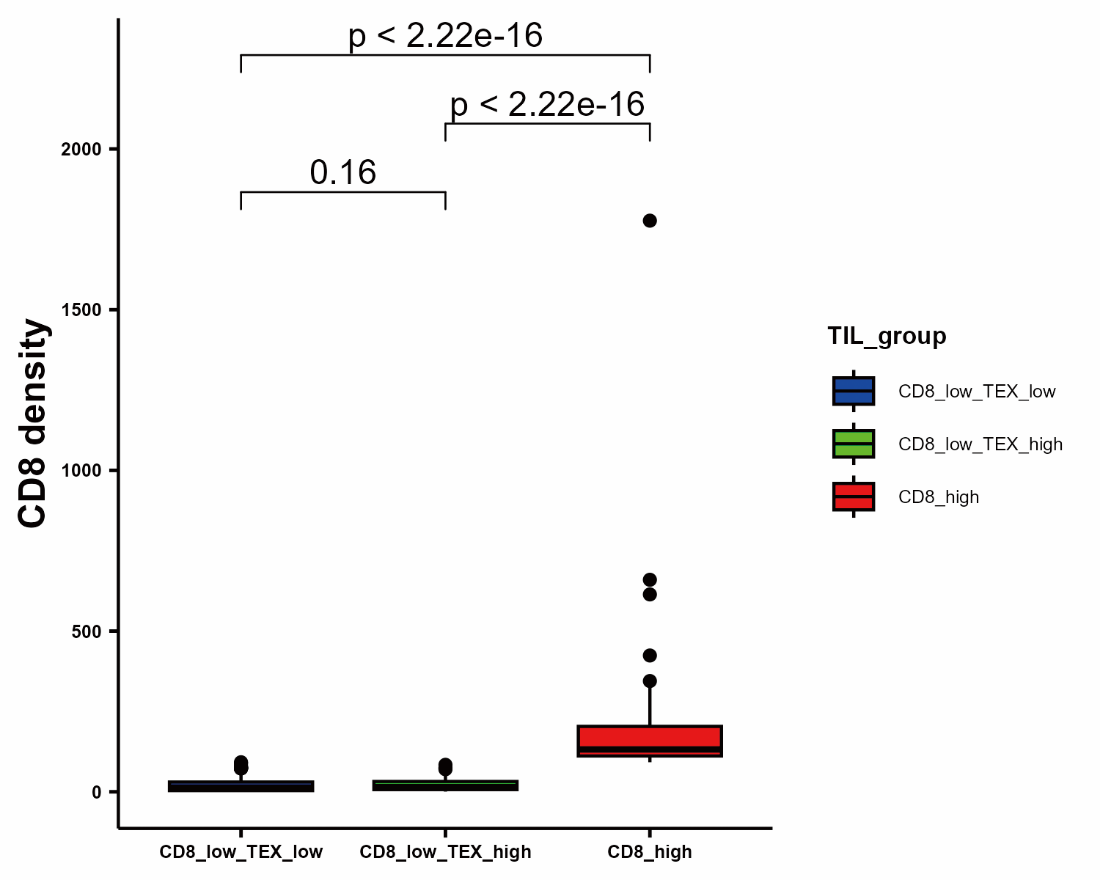


**Supplementary Figure 4.** Box plot for CD8^+^ T cell density according to the tumor-infiltrating lymphocyte (TIL) group based on the CD8^+^ T cell infiltration and terminally exhausted CD8^+^ T cell (Ttex) fraction. ns, *P* > 0.05; ****, *P* ≤ 0.0001 (Wilcoxon’s rank-sum test).

**Supplementary Table 1. List of antibodies and fluorophores used in multiplex immunofluorescence staining.**

| **Stain order** | **Antibody** | | | | **Astra-dye** | | |
| --- | --- | --- | --- | --- | --- | --- | --- |
|  | **Name** | **Vendor** | **Cat#** | **Dilution** | **Name** | **Cat#** | **Dilution** |
| 1 | CD3 | Ventana | 790-4341 | 1:300 | Astra-570 | #C0110 | 1:150 |
| 2 | CD8 | Invitrogen | MA5-13473 | 1:5 | Astra-520 | #C0109 | 1:150 |
| 3 | TCF1/TCF7 | CST | #2203 | 1:500 | Astra-690 | #C0114 | 1:150 |
| 4 | FOXP3 | Abcam | ab20034 | 1:100 | Astra-620 | #C0113 | 1:150 |
| 5 | PD1 | Abcam | ab137132 | 1:200 | Astra-480 | #C0115 | 1:150 |
| 6 | CK | Novus | NBP2-29429 | 1:1000 | Astra-DIG | #C0116 | 1:150 |
|  |  |  |  |  | Astra-780 |  | 1:75 |
| 7 | Astra-DAPI | TheraNovis | #C0106 | 1:1000 |  |  |  |

**Supplementary Table 2. Cell type designation according to protein expression status.**

| **Cell type** | **Marker expression** |
| --- | --- |
| CD3^+^ T cell | CD3^+^ |
| CD8^+^ T cell | CD3^+^CD8^+^ |
| CD4^+^ T cell | CD3^+^CD8^-^ |
| Helper T cell (Thelper) | CD3^+^CD8^-^FOXP3^-^ |
| Regulatory T cell (Treg) | CD3^+^CD8^-^FOXP3^+^ |
| Effector T cell (Teff) | CD3^+^CD8^+^TCF1^-^PD1^-^ |
| Naïve T cell (Tnaïve) | CD3^+^CD8^+^TCF1^+^PD1^-^ |
| Progenitor exhausted T cell (T_pex_) | CD3^+^CD8^+^TCF1^+^PD1^+^ |
| Terminally exhausted T cell (T_tex_) | CD3^+^CD8^+^TCF1^-^PD1^+^ |

**Supplementary Table 3. Mutation frequencies of the 10 most commonly mutated genes in colorectal cancer.**

| **Gene** | **Total**  **(n = 461),**  **No. (%)** | **MSS**  **(n = 428),**  **No. (%)** | **MSI**  **(n = 33),**  **No. (%)** | ***P*** |
| --- | --- | --- | --- | --- |
| *APC* | 329 (71.4%) | 312 (72.9%) | 17 (48.5%) | 0.009 |
| *TP53* | 271 (58.8%) | 268 (62.6%) | 3 (9.1%) | < 0.001* |
| *KRAS* | 195 (42.3%) | 180 (42.1%) | 15 (45.5%) | 0.703 |
| *PIK3CA* | 67 (14.5%) | 56 (13.1%) | 11 (33.3%) | 0.001 |
| *SMAD4* | 44 (9.5%) | 38 (8.9%) | 6 (18.2%) | 0.080 |
| *TCF7L2* | 34 (7.4%) | 24 (5.6%) | 10 (30.3%) | < 0.001 |
| *SOX9* | 31 (6.7%) | 24 (5.6%) | 7 (21.2%) | 0.001 |
| *ARID1A* | 30 (6.5%) | 19 (4.4%) | 11 (33.3%) | < 0.001 |
| *BRAF* | 30 (6.5%) | 27 (6.3%) | 3 (9.1%) | 0.465* |
| *FBXW7* | 26 (5.6%) | 23 (5.4%) | 3 (9.1%) | 0.419* |

*Fisher’s exact test.

Abbreviations: MSI: microsatellite instability, MSS: microsatellite stable.

**Supplementary Table 4. CD3^+^ T cell density per mm^2^ according to the 10 most commonly mutated genes in colorectal cancer.**

| **Gene** | **TC** | | | **IM** | | |
| --- | --- | --- | --- | --- | --- | --- |
|  | **WT** | **MT** | ***P*** | **WT** | **MT** | ***P*** |
| *APC* | 235.8±27.5 | 237.6±21.2 | 0.520 | 287.3±30.3 | 277.0±19.2 | 0.660 |
| *TP53* | 268.5±34.7 | 2157±16.3 | 0.221 | 305.2±27.3 | 262.4±19.9 | 0.217 |
| *KRAS* | 244.4±19.6 | 227.2±30.4 | 0.145 | 314.5±23.4 | 233.6±20.8 | 0.061 |
| *PIK3CA* | 245.8±19.9 | 193.2±23.7 | 0.420 | 28.92±18.5 | 232.0±28.8 | 0.516 |
| *SMAD4* | 237.3±18.6 | 235.9±37.8 | 0.534 | 281.0±17.2 | 268.4±49.0 | 0.915 |
| *TCF7L2* | 241.8±18.3 | 178.7±28.7 | 0.622 | 285.5±17.1 | 209.6±43.6 | 0.163 |
| *SOX9* | 239.6±18.3 | 207.9±40.5 | 0.837 | 276.8±16.8 | 314.7±61.9 | 0.309 |
| *ARID1A* | 223.0±13.6 | 413.7±155.5 | 0.529 | 275.4±16.4 | 335.1±76.7 | 0.385 |
| *BRAF* | 236.5±17.3 | 247.3±94.1 | 0.361 | 278.8±16.5 | 295.2±79.1 | 0.966 |
| *FBXW7* | 236.6±17.2 | 245.3±93.5 | 0.505 | 278.2±16.7 | 304.9±66.4 | 0.433 |

Abbreviations: TC, tumor center; IM, invasive margin; WT, wild type; MT, mutant type

**Supplementary Table 5. CD8^+^ T cell density per mm^2^ according to** **10 most commonly mutated genes.**

| **Gene** | **TC** | | | **IM** | | |
| --- | --- | --- | --- | --- | --- | --- |
|  | **WT** | **MT** | ***P*** | **WT** | **MT** | ***P*** |
| *APC* | 49.5±8.3 | 37.3±7.1 | 0.023 | 70.4±10.1 | 59.2±6.2 | 0.210 |
| *TP53* | 55.5±13.0 | 30.5±3.3 | 0.051 | 70.1±9.6 | 56.9±5.9 | 0.185 |
| *KRAS* | 49.4±9.4 | 28.7±3.7 | 0.081 | 75.3±8.3 | 44.8±4.8 | 0.037 |
| *PIK3CA* | 41.4±6.6 | 36.7±6.5 | 0.878 | 64.0±6.0 | 53.5±9.8 | 0.388 |
| *SMAD4* | 39.3±6.2 | 53.2±10.4 | 0.126 | 62.6±5.7 | 58.7±11.4 | 0.715 |
| *TCF7L2* | 41.6±6.1 | 28.4±6.2 | 0.969 | 63.1±5.6 | 51.3±13.2 | 0.325 |
| *SOX9* | 41.2±6.1 | 34.0±7.7 | 0.423 | 63.0±5.6 | 53.1±11.0 | 0.555 |
| *ARID1A* | 35.5±3.5 | 104.6±62.6 | 0.040 | 59.1±4.8 | 101.3±37.3 | 0.048 |
| *BRAF* | 39.4±5.7 | 60.9±28.3 | 0.869 | 62.4±5.5 | 60.0±16.7 | 0.635 |
| *FBXW7* | 36.8±3.4 | 100.4±76.6 | 0.863 | 59.2±4.7 | 112.7±49.5 | 0.383 |

Abbreviations: TC, tumor center; IM, invasive margin; WT, wild type; MT, mutant type

**Supplementary Table 6. Thelper cell density per mm^2^ according to the 10 most commonly mutated genes in colorectal cancer.**

| **Gene** | **TC** | | | **IM** | | |
| --- | --- | --- | --- | --- | --- | --- |
|  | **WT** | **MT** | ***P*** | **WT** | **MT** | ***P*** |
| *APC* | 144.5±17.4 | 167.2±17.6 | 0.887 | 182.8±22.3 | 188.0±14.5 | 0.991 |
| *TP53* | 179.5±28.3 | 148.4±12.4 | 0.306 | 203.1±20.4 | 175.3±15.0 | 0.270 |
| *KRAS* | 153.3±11.7 | 171.5±28.0 | 0.352 | 203.3±16.9 | 164.5±17.2 | 0.139 |
| *PIK3CA* | 167.7±15.9 | 126.7±17.6 | 0.376 | 193.8±14.0 | 149.9±20.2 | 0.396 |
| *SMAD4* | 162.0±14.9 | 151.6±27.3 | 0.635 | 186.8±12.6 | 184.9±43.7 | 0.905 |
| *TCF7L2* | 163.6±14.6 | 129.0±25.9 | 0.817 | 190.5±12.9 | 138.6±28.6 | 0.262 |
| *SOX9* | 162.5±14.5 | 143.8±35.4 | 0.999 | 183.0±12.2 | 228.4±58.4 | 0.359 |
| *ARID1A* | 151.9±9.9 | 274.6±136.6 | 0.932 | 185.4±12.2 | 202.4±60.4 | 0.685 |
| *BRAF* | 161.7±14.1 | 149.8±55.1 | 0.311 | 184.9±12.1 | 214.7±73.2 | 0.783 |
| *FBXW7* | 163.9±14.4 | 115.8±25.9 | 0.411 | 187.8±12.7 | 165.9±33.6 | 0.691 |

Abbreviations: TC, tumor center; IM, invasive margin; WT, wild type; MT, mutant type

**Supplementary Table 7. Treg density per mm^2^ according to the 10 most commonly mutated genes in colorectal cancer.**

| **Gene** | **TC** | | | **IM** | | |
| --- | --- | --- | --- | --- | --- | --- |
|  | **WT** | **MT** | ***P*** | **WT** | **MT** | ***P*** |
| *APC* | 41.8±4.6 | 33.1±2.6 | 0.014 | 34.1±4.1 | 29.7±2.3 | 0.072 |
| *TP53* | 33.5±3.4 | 36.8±3.1 | 0.825 | 32.0±3.3 | 30.2±2.6 | 0.676 |
| *KRAS* | 41.7±3.5 | 27.0±2.6 | 0.005 | 35.9±3.1 | 24.3±2.3 | 0.025 |
| *PIK3CA* | 36.6±2.6 | 29.9±4.4 | 0.395 | 31.4±2.3 | 28.6±4.4 | 0.950 |
| *SMAD4* | 36.0±2.4 | 31.1±7.2 | 0.372 | 31.6±2.2 | 24.8±3.9 | 0.912 |
| *TCF7L2* | 36.6±2.5 | 21.2±4.4 | 0.081 | 31.9±2.1 | 19.6±5.5 | 0.050 |
| *SOX9* | 35.9±2.4 | 30.0±7.5 | 0.237 | 30.7±2.1 | 33.2±6.6 | 0.731 |
| *ARID1A* | 35.6±2.4 | 34.5±6.1 | 0.444 | 30.9±2.1 | 31.3±6.3 | 0.510 |
| *BRAF* | 35.4±2.3 | 36.6±12.7 | 0.654 | 31.6±2.1 | 20.6±4.2 | 0.655 |
| *FBXW7* | 35.9±2.4 | 29.1±7.3 | 0.434 | 31.2±2.1 | 26.3±5.0 | 0.773 |

Abbreviations: TC, tumor center; IM, invasive margin; WT, wild type; MT, mutant type

**Supplementary Table 8. CD8/CD3 ratio according to the 10 most commonly mutated genes in colorectal cancer.**

| **Gene** | **TC** | | | **IM** | | |
| --- | --- | --- | --- | --- | --- | --- |
|  | **WT** | **MT** | ***P*** | **WT** | **MT** | ***P*** |
| *APC* | 0.179±0.014 | 0.131±0.007 | 0.004 | 0.179±0.014 | 0.131±0.007 | 0.004 |
| *TP53* | 0.168±0.012 | 0.128±0.007 | 0.043 | 0.168±0.012 | 0.128±0.007 | 0.043 |
| *KRAS* | 0.151±0.008 | 0.136±0.010 | 0.125 | 0.151±0.008 | 0.136±0.010 | 0.125 |
| *PIK3CA* | 0.140±0.007 | 0.167±0.021 | 0.702 | 0.140±0.007 | 0.167±0.021 | 0.702 |
| *SMAD4* | 0.139±0.006 | 0.196±0.028 | 0.098 | 0.139±0.006 | 0.196±0.028 | 0.098 |
| *TCF7L2* | 0.143±0.007 | 0.133±0.025 | 0.384 | 0.143±0.007 | 0.133±0.025 | 0.384 |
| *SOX9* | 0.120±0.123 | 0.184±0.026 | 0.077 | 0.120±0.123 | 0.184±0.026 | 0.077 |
| *ARID1A* | 0.139±0.006 | 0.216±0.031 | 0.004 | 0.139±0.006 | 0.216±0.031 | 0.004 |
| *BRAF* | 0.142±0.007 | 0.179±0.032 | 0.282 | 0.142±0.007 | 0.179±0.032 | 0.282 |
| *FBXW7* | 0.142±0.006 | 0.184±0.037 | 0.246 | 0.142±0.006 | 0.184±0.037 | 0.246 |

Abbreviations: TC, tumor center; IM, invasive margin; WT, wild type; MT, mutant type

**Supplementary Table 9. Thelper/CD3 ratio according to the 10 most commonly mutated genes in colorectal cancer.**

| **Gene** | **TC** | | | **IM** | | |
| --- | --- | --- | --- | --- | --- | --- |
|  | **WT** | **MT** | ***P*** | **WT** | **MT** | ***P*** |
| *APC* | 0.595±0.019 | 0.674±0.012 | 0.001 | 0.604±0.017 | 0.666±0.012 | 0.005 |
| *TP53* | 0.638±0.017 | 0.662±0.013 | 0.196 | 0.642±0.016 | 0.654±0.013 | 0.642 |
| *KRAS* | 0.631±0.013 | 0.682±0.016 | 0.017 | 0.651±0.010 | 0.618±0.048 | 0.361 |
| *PIK3CA* | 0.653±0.011 | 0.648±0.027 | 0.830 | 0.655±0.010 | 0.622±0.026 | 0.160 |
| *SMAD4* | 0.653±0.011 | 0.643±0.037 | 0.957 | 0.651±0.010 | 0.635±0.032 | 0.573 |
| *TCF7L2* | 0.650±0.011 | 0.689±0.039 | 0.750 | 0.646±0.010 | 0.689±0.037 | 0.278 |
| *SOX9* | 0.650±0.011 | 0.677±0.038 | 0.489 | 0.648±0.010 | 0.666±0.039 | 0.802 |
| *ARID1A* | 0.659±0.011 | 0.567±0.039 | 0.029 | 0.655±0.010 | 0.584±0.035 | 0.100 |
| *BRAF* | 0.654±0.011 | 0.633±0.045 | 0.715 | 0.651±0.010 | 0.619±0.043 | 0.563 |
| *FBXW7* | 0.628±0.011 | 0.634±0.044 | 0.683 | 0.651±0.010 | 0.618±0.048 | 0.361 |

Abbreviations: TC, tumor center; IM, invasive margin; WT, wild type; MT, mutant type

**Supplementary Table 10. Treg/CD3 ratio according to the 10 most commonly mutated genes in colorectal cancer.**

| **Gene** | **TC** | | | **IM** | | |
| --- | --- | --- | --- | --- | --- | --- |
|  | **WT** | **MT** | ***P*** | **WT** | **MT** | ***P*** |
| *APC* | 0.226±0.016 | 0.194±0.009 | 0.078 | 0.183±0.029 | 0.151±0.008 | 0.056 |
| *TP53* | 0.194±0.013 | 0.209±0.010 | 0.217 | 0.160±0.001 | 0.159±0.009 | 0.802 |
| *KRAS* | 0.218±0.011 | 0.182±0.012 | 0.036 | 0.163±0.009 | 0.155±0.010 | 0.683 |
| *PIK3CA* | 0.207±0.009 | 0.184±0.020 | 0.274 | 0.156±0.007 | 0.179±0.019 | 0.191 |
| *SMAD4* | 0.208±0.008 | 0.160±0.027 | 0.034 | 0.160±0.007 | 0.155±0.023 | 0.754 |
| *TCF7L2* | 0.207±0.008 | 0.148±0.022 | 0.073 | 0.163±0.007 | 0.112±0.022 | 0.032 |
| *SOX9* | 0.209±0.009 | 0.139±0.021 | 0.020 | 0.161±0.007 | 0.139±0.024 | 0.355 |
| *ARID1A* | 0.202±0.008 | 0.217±0.032 | 0.733 | 0.160±0.007 | 0.152±0.024 | 0.836 |
| *BRAF* | 0.204±0.008 | 0.189±0.031 | 0.723 | 0.160±0.007 | 0.153±0.033 | 0.627 |
| *FBXW7* | 0.204±0.008 | 0.182±0.031 | 0.510 | 0.161±0.007 | 0.141±0.026 | 0.580 |

Abbreviations: TC, tumor center; IM, invasive margin; WT, wild type; MT, mutant type

**Supplementary Table 11. Mutation frequencies of the five molecular pathways in colorectal cancer.**

| **Pathway** | **Total**  **(n = 461),**  **No. (%)** | **MSS**  **(n = 428),**  **No. (%)** | **MSI**  **(n = 33),**  **No. (%)** | ***P*** |
| --- | --- | --- | --- | --- |
| WNT | 353 (76.6%) | 325 (75.9%) | 28 (84.8%) | 0.244 |
| p53 | 284 (61.6%) | 275 (64.3%) | 9 (27.3%) | < 0.001 |
| RTK | 241 (52.3%) | 222 (51.9%) | 19 (57.6%) | 0.527 |
| PI3K | 81 (17.6%) | 66 (15.4%) | 15 (45.5%) | < 0.001 |
| TGFβ | 65 (14.1%) | 52 (12.1%) | 13 (39.4%) | <0.001 |

Abbreviations: MSI: microsatellite instability, MSS: microsatellite stable.
